# Novel strategy for reusing agricultural mulch film residual by iron modification for arsenic removal in gold-smelting wastewater

**DOI:** 10.3389/fchem.2022.1036726

**Published:** 2022-10-24

**Authors:** Xiaozhuan Zhang, Kejiang Zhao, Xibao Shi, Zhenbang Tian, Zuohua Huang, Liang Zhao

**Affiliations:** ^1^ Henan International Joint Lab of Key Technology in Water Treatment, Key Laboratory of Yellow River and Huai River Water Environmental and Pollution Control, Ministry of Education, School of Environments, Henan Normal University, Xinxiang, Henan, China; ^2^ Henan Institute of Chemistry, Henan Academy of Sciences, Zhengzhou, Henan, China; ^3^ College of Life Sciences, Henan Normal University, Xinxiang, Henan, China

**Keywords:** arsenic, purification and removal, gold-smelting wastewater, adsorption, iron-modified agricultural mulch film residual

## Abstract

In gold-smelting wastewater after the original treatment process of flocculation and precipitation using mainly lime, a mixture of As, Cu, Pb, Mn, Zn, Al, Ni, and Fe existed with an arsenic concentration of 813.07 mg/L and other ions’ concentration at ug/L levels. In this work, a new clean process of mainly adsorption with self-made adsorbent Fe-PE, which was synthesized by loading ferric lignin on agricultural mulch film residual, was investigated to purify and remove arsenic from gold-smelting wastewater. A batch of column experiments was investigated to explore the reaction behavior between wastewater and adsorbent Fe-PE. The results showed while operating the adsorption columns at a pilot scale for 68 days, the arsenic concentration in the effluent was below 0.5 mg/L, and there was no significant change in the concentration of co-existing metal ions, indicating that Fe-PE had a good selective adsorption performance for arsenic in wastewater. Furthermore, Fe-PE did not dissolve and release Fe ions in wastewater, and the whole process could not produce sludge. This work first suggested an efficient and potential application for the purification and removal of arsenic from gold-smelting wastewater with agricultural mulch film residual after chemical modification, which will provide a novel strategy for reusing the agricultural mulch film residual.

## Introduction

Arsenic at higher concentrations in water or land poses a great threat to human health and ecological safety (Lamm and Kruse, 2005; Rodriguez-Lado et al., 2013; Celebi et al., 2014). It is very important that arsenic residues must be strictly restrained to be directly disposed of into the environment. In metallurgical and mining wastewaters (Sekula, 2008; Nazari et al., 2017), arsenic, usually with an extremely high concentration, is one of the main contaminants among other arsenic-associated minerals such as Cu, Fe, Pb, Zn, and Ni (Anderson and Twidwell, 2008; Oishi et al., 2008; Cheng et al., 2009). It is necessary to investigate an efficient process to remove arsenic from metallurgical wastewater in order to meet the environmental legislation.

Iron oxides or oxyhydroxides have a high affinity to arsenic, and the mobility of As is closely correlated with Fe in natural environments (Cances et al., 2005; Jia and Demopoulos, 2005; Jia et al., 2007; Jia and Demopoulos, 2008). Researchers have concluded an adsorption mechanism where As would be bound as a bidentate inner sphere complex with Fe (Ford, 2002; Jia et al., 2006; Gomez et al., 2011; De Klerk et al., 2015). Otherwise, the precipitation as crystalline scorodite (Langmuir et al., 2006; Bluteau and Demopoulos, 2007; Caetano et al., 2009) was reported to offer the advantages of combining a relatively high arsenic content and low release of arsenic in aqueous solutions, but large amounts of neutralizing agents were necessary because of the high alkalinity in the final leaching solution. Therefore, the process of arsenic removal by precipitation is commonly used by the metallurgical and mining industry (De Klerk et al., 2012; Cui et al., 2014; Coudert et al., 2020), mainly due to its lower cost. However, the concentration of residual arsenic is always at the level of mg/L, which is the limit to the technology of precipitation, and most seriously, much sludge with arsenic contamination will be generated, resulting in serious secondary pollution during transportation, storage, and disposal (Lin, 2004; Feng et al., 2017a; Coudert et al., 2020). Focusing on the possible leakage and secondary pollution of arsenic, this work aims to find a new process for arsenic removal in gold-smelting wastewater by adsorption technology using a new adsorbent, expected with no sludge and hazardous solid waste.

Though different adsorbents such as granular activated carbon, activated alumina, biochar, magnetite nanoparticles, and polymeric adsorbents have been proved to be effective in water for arsenic removal, there have been few studies involving the use of agricultural mulch film residual (AMFR) after chem-modification as adsorbents to remove arsenic in wastewater. Because polyethylene film is widely used for the exchange of heat and moisture in agriculture (Liu et al., 2017), AMFR in large amounts is known as one of the agricultural wastes of hard degradation in a short time and could release hazardous substances such as polyethylene particles, plasticizers, or additives into soils and waters during the process of degradation, and it also has the possibility to become microplastics (Zhang et al., 2021). Because of the low-rate recycling (a recycling rate of less than 2/3 each year in China), potentially severe pollution, and plastic-restriction orders by governmental management (MOA, 2017), it is meaningful to investigate a new strategy to reuse AMFR. In our previous work (Zhang et al., 2022), ferric lignin has been loaded on the polyethylene film, and AMFR will also be promisingly modified by ferric lignin because of the same composition as the original polyethylene film. But before modification, AMFR must be pretreated, for example, cleaning to move the pollutants adhered to the surface of AMFR.

In this work, we monitored a kind of gold-smelting wastewater, after the original treatment of flocculation and precipitation using mainly lime; a mixture of arsenic, copper, lead, manganese, zinc, aluminum, nickel, cadmium, and iron existed with an arsenic concentration of 813.07 mg/L and other ions’ concentration was at the level of ug/L. AMFR would be loaded with ferric lignin after chemical modification and used in the process of arsenic removal in this wastewater. A batch of column experiments was investigated to explore the reaction behavior between wastewater and the adsorbent, and the objective of this work was to investigate an integrated process for arsenic purification from gold-smelting wastewater, which may have potential application for arsenic removal in all gold-smelting wastewaters after the precipitation process using mainly lime as pretreatment.

## Experimental

### Materials and reagents

Sodium lignosulphonate, ferrous sulfate heptahydrate, sodium sulfite anhydrous, sodium hydroxide, and ethanol absolute were all in reagent grade and purchased from Shanghai Macklin Biochemical Co., Ltd., China.

Different specifications of plexiglass ion-exchange columns were ordered from Zhengzhou Glass Factory, China. The column with a diameter of 50 mm and a length of 500 mm was used for a lab-scale experiment, and the column with a diameter of 300 mm and a length of 1500 mm was used for a pilot-scale experiment. The centrifugal pump, peristaltic pump, and flowmeter were purchased from Jiangsu Pump Industry, Co., Ltd. Taizhou, China. The normal window gauze, bought from a local grocery store, was applied as the supporting material. The de-ionized water was used for dilution, and 1 M of HCl or NaOH was used for adjusting the pH of the solution.

The gold-smelting wastewater after the original treatment process of flocculation and precipitation using mainly lime, was from a local company smelting precious metals such as gold and copper. Table 1 shows the chemical composition of the wastewater during the period of observation. In view of the potentially toxic substances, during the process of handling the wastewater, CAUTION in the Supplementary Materials must be obeyed.

**TABLE 1:** Composition of the gold-smelting wastewater after the original treatment process of flocculation and precipitation using mainly lime (mg/L).

Element	As	Mn	K	Ni	Zn	Fe	Ba	Al	Cu	Pb	SO_4_^2−^	NO_3_^−^	CO_3_^2−^
Concentration	813.07	0.428	0.332	0.205	0.271	0.167	0.065	0.044	0.039	0.011	1.013	0.586	3.261

### Method for preparing arsenic-removal adsorbent

The adsorbent named Fe-PE used by us in this work was prepared by loading ferric lignin on AMFR. The synthesis route of Fe-PE is shown in Figure S1. AMFR exposed for 6 months in the farmland with growing romaine lettuce plants was manually fetched without pulling too much, and the thickness of AMFR was 0.012 mm with a tensile resistance strength of 1.77 N. After being cleaned by watersteam with pressure and HCl or NaOH solution to remove the surface pollutants, cleaned AMFR was dried at a room temperature of 25°C–30°C and immersed into 1 M NaOH solution in an ultrasonic reactor for 12 h at 70°C. Solutions of sodium lignosulphonate, ferrous sulfate heptahydrate, and sodium sulfite anhydrous were allowed to react to get ferric lignin by adjusting the pH using 0.1 M of NaOH. Then, AMFR and ferric lignin solution were put into a water-bath kettle at 80°C for 6–8 h. After that, AMFR was dried in an air oven at 100°C–140°C. In addition to the soil, mud, and plant residues, especially in the process of cleaning the AMFR, 1.007 mg/L of chlorantraniliprole and 0.001 mg/L of phoxim were detected and measured by GC-MS/MS in the wastewater after the cleaning process, due to the insecticides used in the growth of romaine lettuce plants, so the wastewater after the cleaning process must be collected.

### Adsorption experiments using Fe-PE for arsenic removal on the lab scale

The arsenic-bearing wastewater was collected from the outlet after the process of precipitation, and the pH was 8.62∼8.89. The total arsenic concentration was 813.07 mg/L with 68.7% of As (V) and 31.3% of As (III). The size of the column with a volume of 0.981 L was 50 mm diameter and 500 mm length. A peristaltic pump was used to supply impetus. The load density (LD) was calculated according to the following equation: LD = the amount of Fe-PE (g)/the volume of the column (L).

Static adsorption experiments were used to determine the adsorption capacity of the Fe-PE. The arsenic-bearing wastewater was diluted into different concentrations of 8.03, 16.23, 32.35, 81.60, 161.9, 405.2, and 813.07 mg/L. Different mass of 5 g, 10 g, and 20 g of Fe-PE were, respectively, immersed in the wastewater of 1 L in a plastic bucket for 12 h. The Fe-PE was wrapped in a window gauze before to be used.

### Methods used to set up the column experiment

A batch of column experiments was conducted to investigate the performance of Fe-PE to remove arsenic from the wastewater. Fe-PE was wrapped in the window gauze and filled into the plexiglass ion-exchange column. The wastewater was pumped into the column from the bottom to the top. The basic flow chart of the adsorption process is shown in Figure S2.

### Characterizations and testing

FT-IR spectra were collected on a spectrometer in the range of 400–4,000 cm^−1^ (jx20112184, PerkinElmer, United States). The pH value of liquid samples was measured using a PHS-3E pH meter (Shanghai, LeiCi, China). Element concentrations of liquid samples were measured using an ICP-OES spectrometer (PQ-900, Analytik Jena, Germany) or ICP-MS (jx20110281, PE, United States). An FESEM (Quanta FEG250, Thermo Fisher Scientific, United States) was used to scan the surface of the adsorbent and further confirm the distribution of elements.

## Results and discussion

### FT-IR and SEM analyses of the prepared adsorbent Fe-PE

**FIGURE 1:**
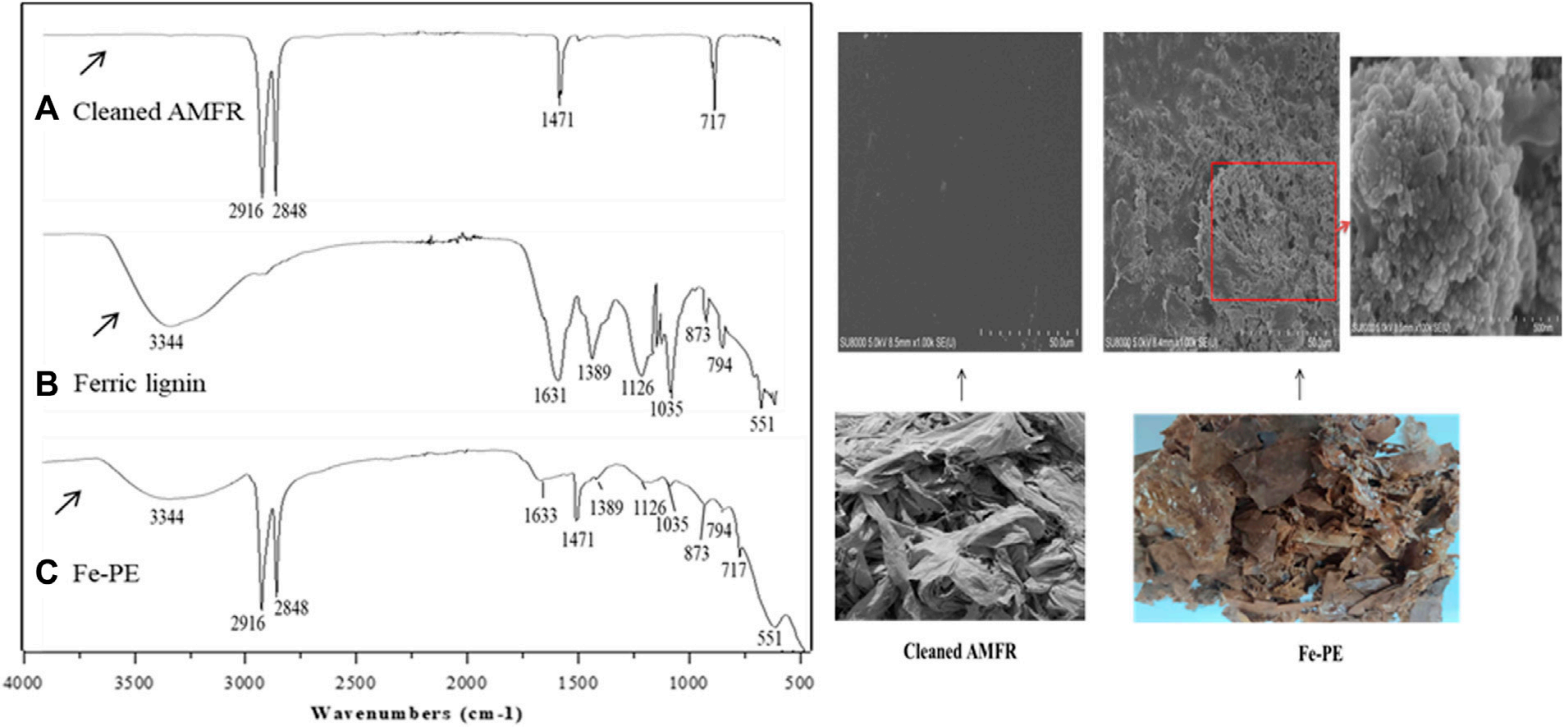
FTIR spectra of **(A)** cleaned AMFR, **(B)** ferric lignin, and **(C)** Fe-PE and SEM images and photographs of cleaned AMFR and Fe-PE.

An FT-IR spectrum was used to predict and confirm the reaction behavior between cleaned AMFR and ferric lignin. Figure 1 shows the spectra of cleaned AMFR (Figure 1A), ferric lignin prepared by us (Figure 1B), and Fe-PE (Figure 1C). As shown in Figure 1A, adsorption peaks at 2,916 cm^−1^, 2848 cm^−1^, 1471 cm^−1^, and 717 cm^−1^ represent the antisymmetric stretching, symmetrical stretching, bending, and rocking vibration of -CH_2_, respectively, which are the four characteristic adsorption peaks of polyethylene (Weng, 2010). The IR spectra of cleaned AMFR showed that there was no other obvious characteristic adsorption peaks except that of polyethylene, indicating that there was no other substance on the cleaned AMFR. As shown in Figure 1B, there were adsorption peaks at 3,344, 1,631, 1,389, 1,126, 1,035, 873, 794, and 551 cm^−1^ of ferric lignin. As shown in Figure 1C, compared with Figure 1A, the spectra showed that the absorption peaks at 2916 cm^−1^, 2848 cm^−1^, 1471 cm^−1^, and 717 cm^−1^ were obviously weakened, and new adsorption peaks at 3,344, 1,633, 1,389, 1,126, 1,035, 873, 794, and 551 cm^−1^ appeared. Compared with Figure 1B, except for the characteristic adsorption peaks of polyethylene, there were same adsorption peaks on Fe-PE as those on ferric lignin, indicating that ferric lignin has been loaded on AMFR. Furthermore, the total content of the Fe element on Fe-PE was determined using an ICP-OES spectrometer after Fe-PE was digested with the mixed acid of HNO_3_–HClO_4_–HF, and 263.68 mg (on average) of Fe element on 1 g of Fe-PE could be examined.

Figure 1 also shows the SEM images and photographs of Fe-PE and cleaned AMFR. It showed that there were many flower-like particles on Fe-PE, compared to that of AMFR. The images also showed that the distribution of the particle size was about 500 nm and the morphology was uniform, indicating that the reaction was sufficient and moderate.

### Adsorption behaviors of arsenic removal from gold-smelting wastewater by Fe-PE on the lab scale

Figure S3 shows the arsenic adsorption capacity of Fe-PE. There is a trend that higher solution concentration results in increasing adsorption capacity, but there is no expected increasing trend when using more Fe-PE, probably due to the effect of the As/Fe ratio and effective adsorption sites on the surface of Fe-PE during the process of adsorption. From Figure S3, the higher adsorption capacity of 44.73 mg/g for arsenic could be calculated, while using 10 g of Fe-PE in 1 L of wastewater with arsenic concentration of 813.07 mg/L.

**FIGURE 2:**
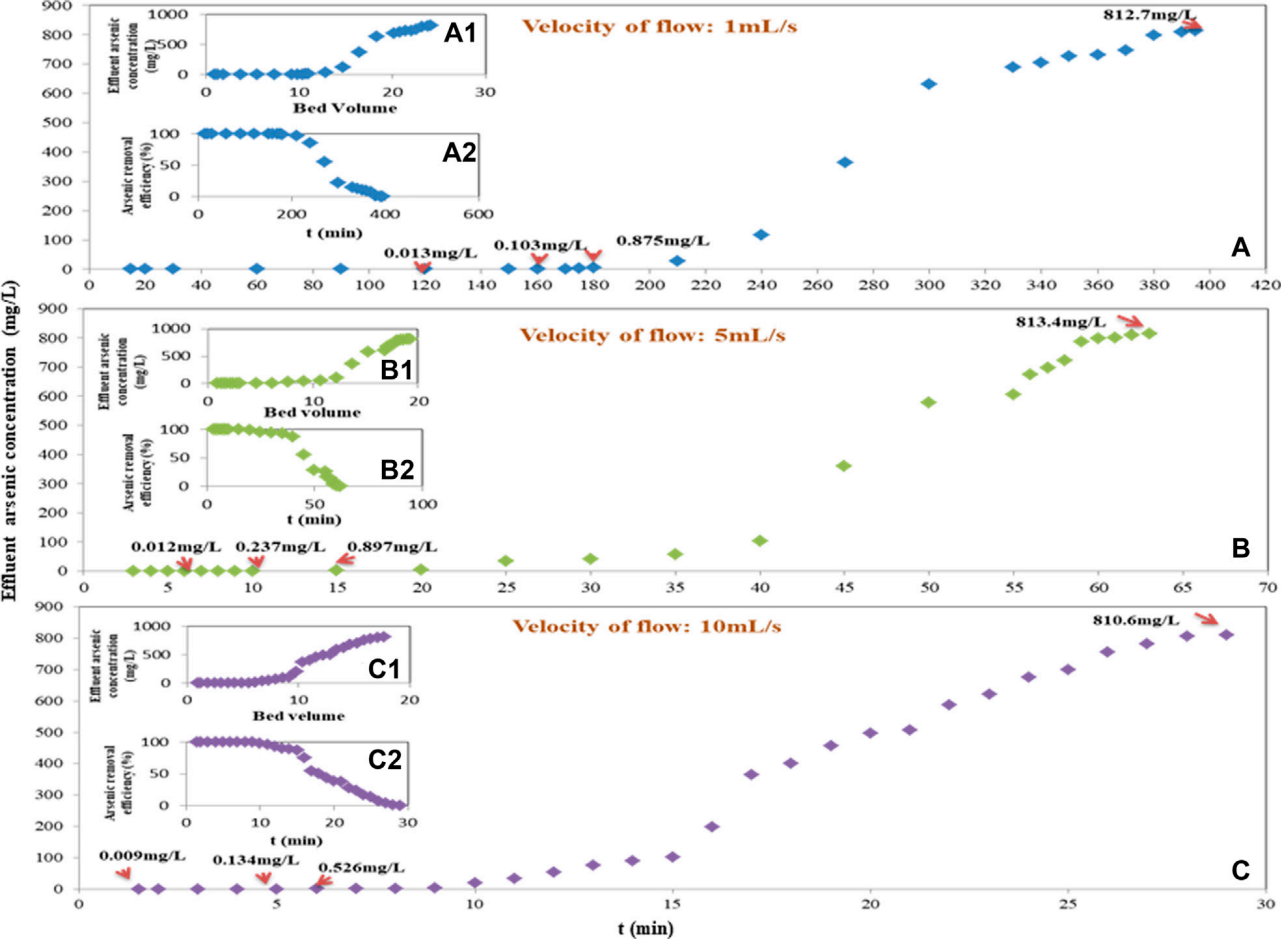
Adsorption column experiments on the lab scale [A, B, and C curve of breakthrough. **(A1)**, **(B1)**, and **(C1)** curve of bed volume. **(A2)**, **(B2)**, and **(C2)** curve of arsenic-removal efficiency. The velocity of flow was 1.0, 5.0, and 10.0 ml/s].

Based on the arsenic adsorption capacity of Fe-PE and the volume of the column, while the adsorption column was full of Fe-PE and window gauze, the load density (LD) should be 360∼400 g/L except for the weight of the window gauze. Also, the lower operating pressure must be taken into consideration. Figure 2 shows the adsorption performance of Fe-PE in column experiments in the laboratory. Figure 2A shows the curve of the breakthrough, Figure 2A1 shows the curve of the bed volume, and Figure 2A2 shows the curve of arsenic removal efficiency, when the velocity of the flow was 1 ml/s. Figure 2B shows the curve of the breakthrough, Figure 2B1 shows the curve of the bed volume, and Figure 2B2 shows the curve of arsenic removal efficiency, when the velocity of the flow was 5 ml/s. Figure 2C shows the curve of the breakthrough, Figure 2C1 shows the curve of the bed volume, and Figure 2C2 shows the curve of arsenic removal efficiency, when the velocity of the flow was 10 ml/s.

The empty bed contact time (EBCT) of the column was investigated and analyzed by different values of the flow velocity. When the velocity of the flow was 1, 5, and 10 ml/s, the EBCT was 981, 196.2, and 98 s, respectively. With the longer EBCT, as shown in Figure 2A, the arsenic concentration in the effluent was kept below 0.013 mg/L within 120 min, and 99% of arsenic could be removed when the column was operated for 180 min. With the shorter EBCT, as shown in Figure 2C, the arsenic concentration in the effluent was kept below 0.013 mg/L within 1 min, and 99% of arsenic was removed when the column was operated for 9 min. The adsorption efficiency decreased as the velocity increased with a correspondingly shorter EBCT. Within the scope of experimental investigation, the optimum velocity was 1 ml/s with an EBCT of 981 s, and the bed volume was 24.15 after the effluent arsenic concentration exceeded 813.07 mg/L. Also, when the flow velocity was 5 or 10 ml/s, the bed volume was 19.27 or 17.73, respectively. Figure 2 shows that the breakthrough time and arsenic removal efficiency increased with the increasing EBCT. Furthermore, based on the measured arsenic concentration (on average) of all effluent water during the time period at different flow velocities, the loading mass of Fe-PE in the column, and the column volume, the arsenic adsorption capacity for every column experiment could be 65.63, 53.46, and 49.87 mg/g at the velocity of 1, 5, and 10 ml/s, respectively, indicating a higher arsenic adsorption capacity with an increasing EBCT. The arsenic adsorption capacity calculated from the column experiment was a little higher than that calculated in the static experiment in this work and also a little higher than that calculated in other’s work (Feng et al., 2017a; Feng et al., 2017b) using nano Fe_3_O_4_@SiO_2_@TiO_2_ or γ-Fe_2_O_3_@ZrO_2_ with the total arsenic adsorption capacity of 21.3 or 42.3 mg/g during the process of removing arsenic from simulated process water of a cyanide gold leach plant, probably due to the faster mass transfer and larger contact area between arsenic and Fe-PE in the column process with a proper EBCT. The results showed again that effective and sufficient contact between the adsorbents and contaminants was essential in the process of adsorption (Neumann et al., 2013; Shakya and Ghosh, 2018).

The aforementioned experiments showed that arsenic could be promisingly removed by Fe-PE to meet drinking-water standards (below 0.01 mg/L) or discharging standards for arsenic in industrial wastewater (below 0.5 mg/L) with a proper velocity flow and EBCT.

### Performance of Fe-PE for arsenic removal at varying initial arsenic concentrations with different pH values

**FIGURE 3:**
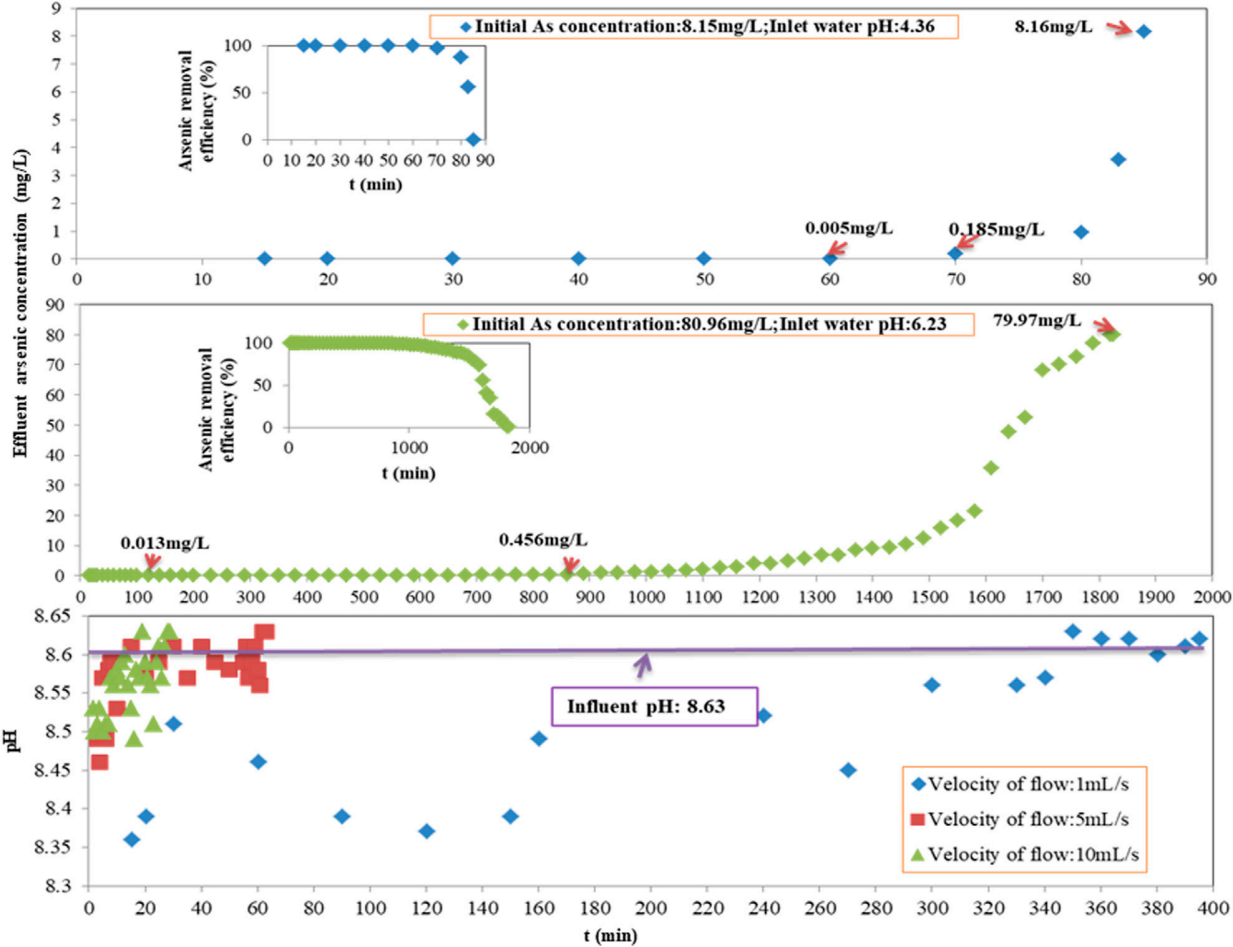
Performance evaluation of Fe-PE for arsenic removal at an initial arsenic concentration of 8.15 mg/L with pH = 4.36 and 80.96 mg/L with pH = 6.23; and the effluent pH of the adsorption column with time at different velocities of the flow.

In the process of column adsorption, varying initial arsenic concentrations of 8.15 mg/L (with a pH of 4.36 adjusted by HCl), 80.96 mg/L (with a pH of 6.23 adjusted by HCl), and 813.07 mg/L (with a pH of 8.63, the original wastewater) were applied after the wastewater was diluted. The arsenic removal performance of Fe-PE is shown in Figure 3. The operating conditions were as follows: the EBCT was 981 s at a flow velocity of 1 ml/s, and the LD of Fe-PE was 370 g/L except for the weight of the window gauze.

Figures 2, 3 show that under the same operating conditions, inlet wastewater with a lower initial arsenic concentration resulted in a better adsorption performance of Fe-PE than that with a higher initial arsenic concentration, due to the adsorption capacity of the adsorbents. More interestingly, there occurred an exception when the concentration of inlet wastewater was 8.15 mg/L. Researchers (Antelo et al., 2005; Capobianco et al., 2020; Fan et al., 2020) have revealed that the solution pH has an important effect on adsorption performance because of different arsenic species under different pH conditions as well as the effects of H^+^ or OH^−^. But in this work, Fe-PE was prepared under alkaline conditions, so acidic solutions with more H^+^ may have an effect on the dissolution of Fe ions from Fe-PE and then on the performance of Fe-PE for arsenic removal. To verify this, 1 g of Fe-PE was immersed into the deionized water with HCl or NaOH, the pH was adjusted to 2.13, 4.01, 6.15, 8.07, and 10.05, and the concentration of total Fe ions was measured using an ICP-OES spectrometer. Simultaneously, another 1 g of Fe-PE was put into 5 mg/L of arsenic solutions with HCl or NaOH, the pH was adjusted to 2.05, 4.17, 6.36, 8.17, and 10.19, and the concentration of total arsenic was measured using an ICP-OES or ICP-MS spectrometer. The results are shown in Figure S4. Under acidic conditions, Fe ions were detected in deionized water. The lower the pH values, the more the Fe ions, and it showed Fe ions were in the solution from Fe-PE because of the reaction between Fe oxides and H^+^. Correspondingly, the arsenic concentration in the solution was higher during the process of arsenic removal by Fe-PE at pH = 2.05 or 4.17, resulting in a lower arsenic removal efficiency. On the contrary, under neutral and alkaline conditions, almost no iron ions were examined, resulting in a higher arsenic removal efficiency correspondingly during the process of arsenic removal by Fe-PE at pH = 6.36 or 8.17 or 10.19. However, it was not that the higher the alkalinity was, the more favorable the adsorption was because a large amount of OH^−^ under strong alkaline conditions will compete with arsenic ions to occupy the effective adsorption sites on Fe-PE. As shown in Figure S4, during the process of arsenic removal by Fe-PE, the optimum pH value of the inlet should be approximately 8.00.

Also, this work investigated the pH of the effluent of the adsorption column. The result is shown in Figure 3. At a flow rate of 1 ml/s, the pH of the effluent decreased slightly with time. When the flow rate increased, the pH of the effluent did not change significantly with time, especially when the flow rate reached 10 ml/s. Iron-based adsorbents may decrease the pH of water because of H^+^ releasing from the reaction between Fe and As during the process of the adsorption. The experimental results of detecting the pH value of the effluent of the adsorption column in this work were consistent with these conclusions, but applying Fe-PE for arsenic removal will not significantly change the pH value of the raw water. In order to further verify this result, we reduced the flow rate to 0.1 ml/s to ensure full contact and reaction of Fe and As and observed the change in the effluent pH value with time. When the experiment reached the point of breakthrough, the lowest pH value of the effluent from the adsorption column was 8.36, which was still within the optimal range of the pH value in the process of arsenic adsorption by Fe-PE (data not shown). Furthermore, Figures 2, 3 reveal that the pH value of the effluent of the adsorption column was correlated with the arsenic-removal performance of Fe-PE at the same time period, in which the pH of the effluent would decrease slightly during the period of high adsorption efficiency and was almost the same as that of the inlet at the point of breakthrough.

### Effect of backflushing with NaOH on the performance of Fe-PE for arsenic removal

**FIGURE 4:**
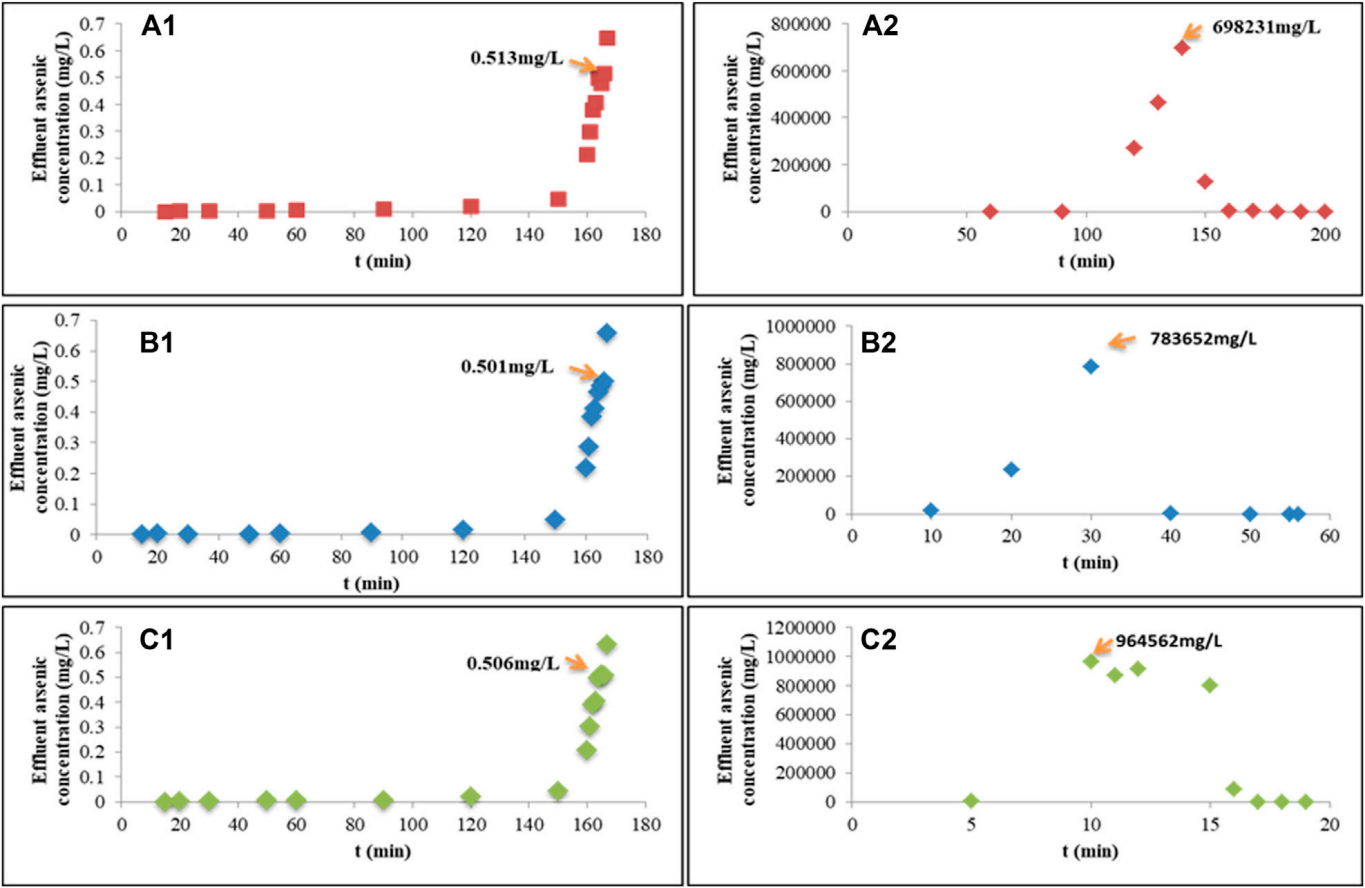
Adsorption breakthrough curves at the breakthrough point of 0.5 mg/L and backflushing curves using 1 M of NaOH with different velocities of the flow. **(A1)** Adsorption curves and **(A2)** the corresponding backflushing curve with an inlet velocity of flow = 0.1 ml/s; **(B1)** adsorption curves and **(B2)** the corresponding backflushing curve with an inlet velocity of flow = 0.5 ml/s; **(C1)** adsorption curves and **(C2)** the corresponding backflushing curve with an inlet velocity of flow = 1 ml/s.

The arsenic concentration to meet the discharging standards for arsenic in industrial wastewater was below 0.5 mg/L. Backflushing began while the arsenic concentration in the outlet of the adsorption column was greater than 0.5 mg/L. The velocity of flow for adsorption was 1 ml/s, the concentration of inlet wastewater was 813.07 mg/L, the diameter of the column was 50 mm and the length was 500 mm, and the LD of Fe-PE was 371 g/L. The direction of the water inlet and outlet during backflushing is opposite to that of the adsorption process. The end of the backflushing process using NaOH was determined by detecting the concentration of arsenic in the backflushing water. The effect of 0.1 M, 0.5 M, 1 M, 5 M, and 10 M of NaOH has been studied, and the results (data not shown) showed that 1 M of NaOH could have a better backflushing performance. So 1 M of NaOH was applied in the process of backflushing. Figure 4 shows the backflushing performance at different velocities of the flow. The results showed that applying NaOH for backflushing could desorb arsenic from Fe-PE because OH^−^ could compete with arsenate or arsenite ions to occupy the effective adsorption sites on Fe-PE, so that arsenic ions could enter into the solution. Usually, in the backflushing process, we hope that the flushing speed is not too slow, the efficiency of arsenic desorption is higher, and a small amount of desorption solution with a higher concentration of arsenic can be gathered. Figure 4 shows that the flow rate of NaOH had significant effects on backflushing. When the flow rate of NaOH was 0.1 ml/s, the arsenic concentration in the desorption solution could reach 698,231 mg/L. When the flow rate of NaOH was 0.5 ml/s, the arsenic concentration in the desorption solution could reach 783652 mg/L. When the flow rate of NaOH was 1 ml/s, a higher arsenic concentration in the desorption solution could reach 964,562 mg/L. Furthermore, when the arsenic concentration in the outlet was at the ppb level and the backflushing ended, the arsenic desorption solutions gathered in three groups were 1.2 L, 1.7 L, and 1.1 L with average arsenic concentrations of 6681.4, 4337.1, and 5469.0 mg/L, respectively, and the backflushing lasted for 200 min, 56 min, and 19 min, respectively. The total arsenic (in mass) eluted from the columns in three groups was 8017.7, 7373.1, and 6015.9 g, respectively. Compared with the total arsenic (in mass) adsorbed on Fe-PE in columns in three groups, which was 8049.4, 8046.1, and 7997.3 g, 99.6%, 91.6%, and 75.2% of arsenic could be eluted, respectively, from the columns in three groups. In order to judge which experimental conditions were more suitable for the backflushing, a further experiment was conducted to evaluate the adsorption performance for arsenic removal after backflushing the adsorption column. The operation process was as follows: the adsorption column after backflushing was used for the next adsorption experiment. Figure S5 shows the results of the adsorption performance before and after backflushing. Columns 1, 2, and 3 represented the experiments in three adsorption columns operated simultaneously. Under the same operating conditions, before backflushing, three columns reached the breakthrough point (0.5 mg/L) after 166 min. Then, after columns 1, 2, and 3 were backflushed using 1 M of NaOH at 0.1, 0.5, and 1 ml/s, respectively, the second adsorption began. Compared with the first adsorption, although the time taken to reach the adsorption breakthrough was earlier, the adsorption columns after backflushing were still effective for arsenic removal, indicating that backflushing with 1 M of NaOH could regenerate Fe-PE. Figure S5 also shows that with the lower flow rate, the time taken to reach the adsorption breakthrough in the column after backflushing was closer to the time in the first adsorption, which indicated that the lower flow rate of NaOH solution resulted in efficient desorption of arsenic from Fe-PE.

### Treatment of gold-smelting wastewater by Fe-PE in the company on the pilot scale

**FIGURE 5:**
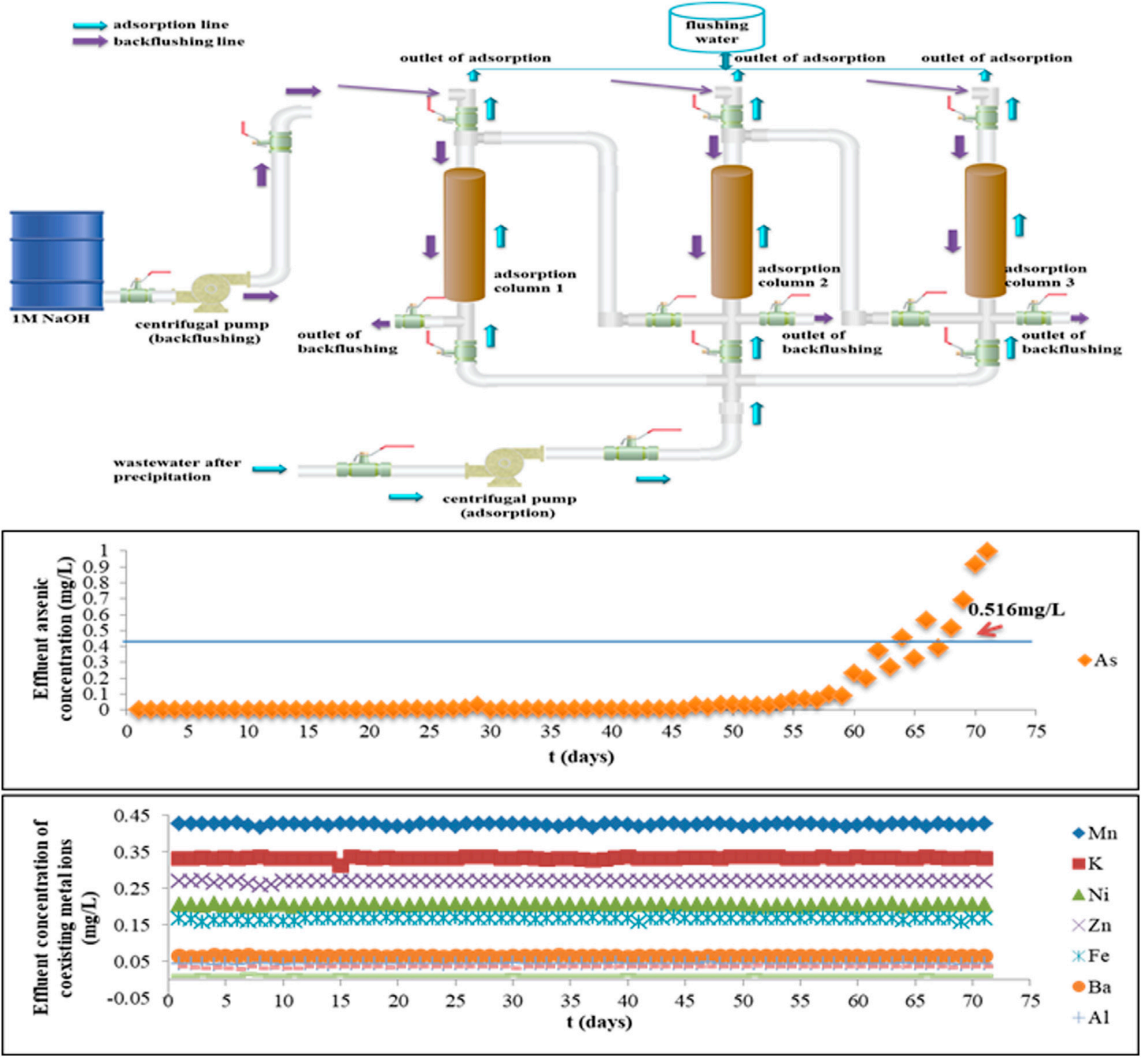
Pilot-scale schematic diagram and the performance evaluation of the adsorption and backflushing system for arsenic removal by Fe-PE.

Based on the overall consideration of the performance of Fe-PE for arsenic removal on the lab scale, a group of three adsorption columns in series controlled by valves was used for pilot experiments. Figure 5 shows the pilot scale schematic diagram of the adsorption and backflushing system. When pilot equipment started, columns 1 and 2 were used for adsorption, and column 3 was on standby. When column 1 was saturated, the adsorption was switched to columns 2 and 3, and column 1 was backflushed. Part of the treated water was used to flush the adsorption column after backflushing, in order to discharge the residual NaOH to avoid the effect of OH^−^ on the adsorption. Therefore, while switching the processes of adsorption and backflushing, continuous operation started. Samples were taken every 30 min, and samples taken during the day were tested at night, and samples taken at night were tested the next day.

After precipitation with lime, the temperature of wastewater was 36°C–38°C, pH was 8.62–8.89, and arsenic concentration was 813.07 mg/L. According to the adsorption capacity of Fe-PE for arsenic and the influence of flow rate and EBCT on the adsorption process, as well as the point of breakthrough (0.5 mg/L), the experimental parameters selected for the pilot-scale experiment were as follows: the diameter of the column was 300 mm and the length was 1500 mm, the LD of Fe-PE was 373 g/L, and the velocity of the flow for adsorption was 10 L/min to ensure that the EBCT was not lower than 10 min.

The performance of arsenic removal by Fe-PE from gold-smelting wastewater in the company on the pilot scale is shown in Figure 5. Simultaneously, the performance of pilot equipment to arsenic and other coexisting metal ions was evaluated. There was no significant change in the concentration of coexisting metal ions in the effluent, indicating that the adsorption column did not work with the removal of coexisting metal ions, and the adsorbent had good selective adsorption performance for arsenic in wastewater and also indicating that Fe-PE did not dissolve and release Fe ions in wastewater. Within 68 days of the operation of the adsorption column, the concentration of arsenic in the effluent was below 0.5 mg/L. The pH of effluent water was between 8.36 and 8.65 during the entire period of operation (data not shown). After the column adsorption, the concentration of SO_4_^2-^, NO_3_^−^, and CO_3_^2-^ in effluent water was kept almost the same as before. Thus, it could be concluded that the adsorption column with Fe-PE could remove arsenic efficiently with an adequate EBCT of not less than 10 min in this experiment.

### Characterizing the adsorbent Fe-PE after adsorption saturation

**FIGURE 6:**
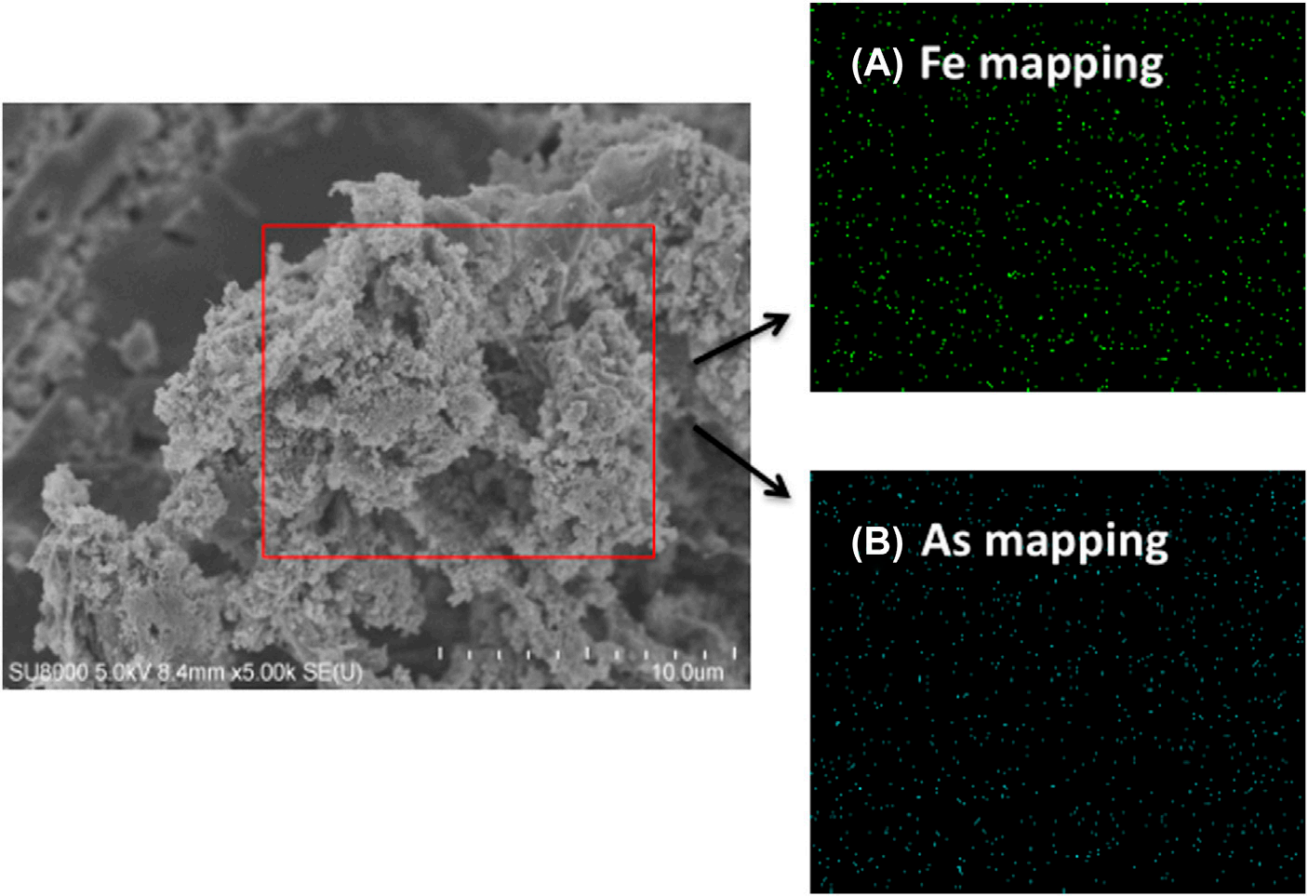
FESEM micrograph and elemental mapping of Fe-PE after adsorption. **(A)** Mapping of iron element and **(B)** mapping of arsenic element.

Figure 6 shows FESEM images of the surface of Fe-PE after arsenic adsorption in the wastewater, and the presence of small and uniform granular structures could be clearly seen. The elemental distribution on the surface of Fe-PE was further confirmed by elemental mapping. Figure 6 shows the mapping of the encircled portion, which showed the uniform distribution and presence of a substantial amount of iron and arsenic on the surface of Fe-PE. Elemental mapping of individual elements clearly indicated the higher content of iron and arsenic.

## Conclusion

In this study, the process of purification and removal of arsenic in gold-smelting wastewater was conducted by column experiments with Fe-PE. Whether it was a laboratory test or a pilot test, Fe-PE had good purification performance for arsenic. Within 68 days of operating the adsorption column on the pilot scale, the concentration of arsenic in the effluent was below 0.5 mg/L, and there was no significant change in the concentration of coexisting metal ions in the effluent, indicating that the adsorbent had good selective adsorption performance for arsenic in wastewater. There was a high content of iron and arsenic on the surface of the saturated Fe-PE tested using an FESEM by elemental mapping of individual elements. Furthermore, compared with the conventional process, the promising environmental advantages of this work are the following: the higher concentration of arsenic in backwashed water obtained in this work deserves the refinement of arsenic compounds by precipitating or recrystallization. Fe-PE with arsenic adsorption could be pyrolyzed in which the ash could be leached by acid or alkali to refine the arsenic compound, and the exhaust gas with higher temperature could be used for the heater and could be purified using a biological filter or a functional fibrous filter. Therefore, it could be concluded from the present findings that arsenic could be removed from gold-smelting wastewater by using agricultural exhausted polyethylene film loaded with ferric lignin at an optimal empty bed contact time, and the adsorption and backflushing system on the pilot scale could make arsenic concentration possible to meet the discharge standards for arsenic (below 0.5 mg/L) in industrial wastewater, with no sludge being produced.

However, the pretreatment of cleaning the AMFR before modification and loading the Fe-PE into columns was a laborious and time-consuming job by manual operation, so the machinery promotion by automatic or semi-automatic operation may be suitable in the future.

## Data Availability

The original contributions presented in the study are included in the article/Supplementary Material; further inquiries can be directed to the corresponding author.
