# Supplementary material for: Novel strategy for reusing agricultural mulch film residual by iron modification for arsenic removal in gold-smelting wastewater
Source: Front Chem. 2022 Oct 24;10:1036726. doi: 10.3389/fchem.2022.1036726 (PMC9638166; doi:10.3389/fchem.2022.1036726)
Supplement: Supplementary file 1 [file Presentation1.pdf]

Supplementary material

Novel Strategy of Reusing Agricultural Mulch Film Residual by Iron-Modification for Arsenic Removal in Gold Smelting Wastewater

Xiaozhuan Zhang[1a,*, †], Kejiang Zhao[1a,2, †], Xibao Shi[1b, †], Zhenbang Tian[2], Zuohua Huang[2], Liang Zhao[2]

[1a] Henan International Joint Lab of Key Technology in Water Treatment, Key Laboratory of Yellow River and Huai River Water Environmental and Pollution Control, Ministry of Education, School of Environments, Henan Normal University, Xinxiang 453007, Henan, China

[1b] College of Life Sciences, Henan Normal University, Xinxiang453007, Henan, China

[2] Henan Institute of Chemistry, Henan Academy of Sciences, Zhengzhou 450002, Henan, China

* Corresponding author at: School of Environments, Henan Normal University, Xinxiang 453007, Henan, China. E-mail address: zhangxiaozhuan0103@126.com;

[†] These authors have contributed equally to this work and share first authorship.

Pictures of agricultural mulch film residual used in this work:

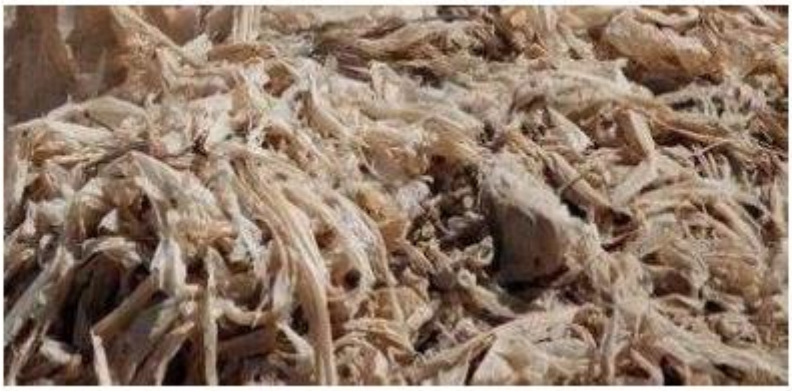

Fig.S1 The synthesis route of Fe-PE

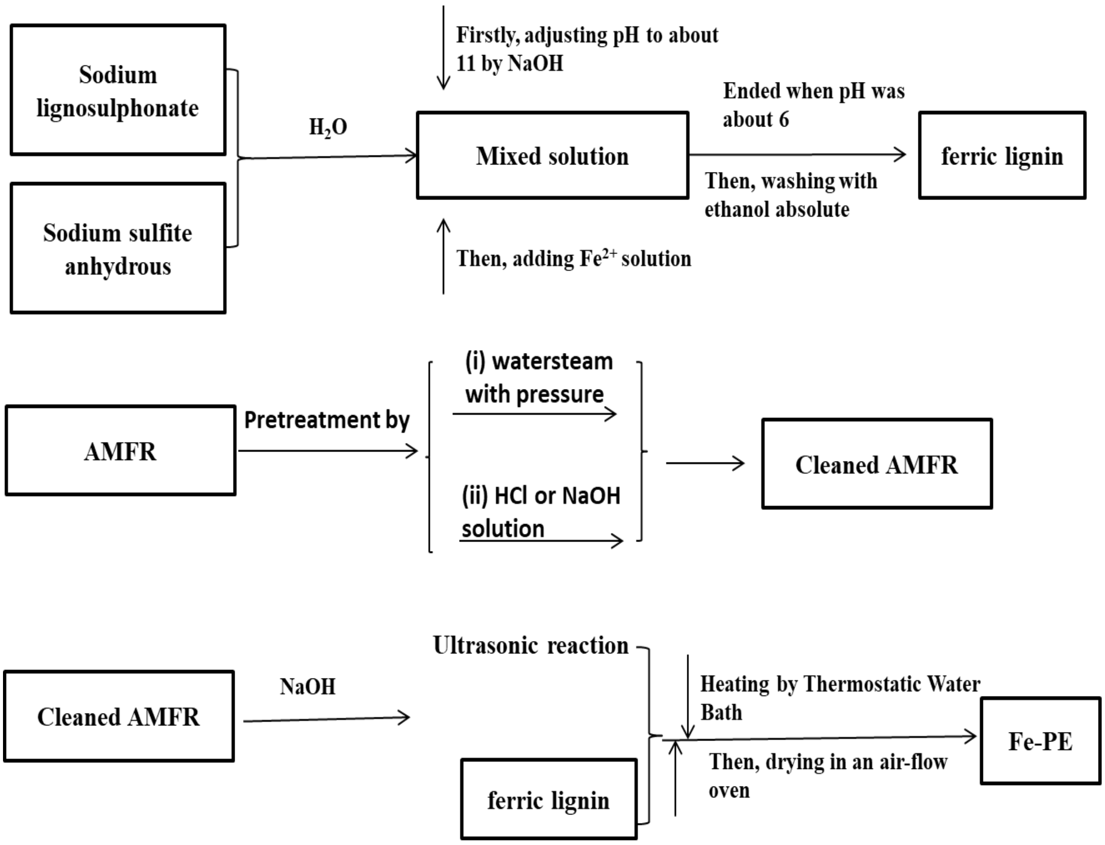

Fig.S2 Basic schematic diagram of adsorption column loaded with Fe-PE.

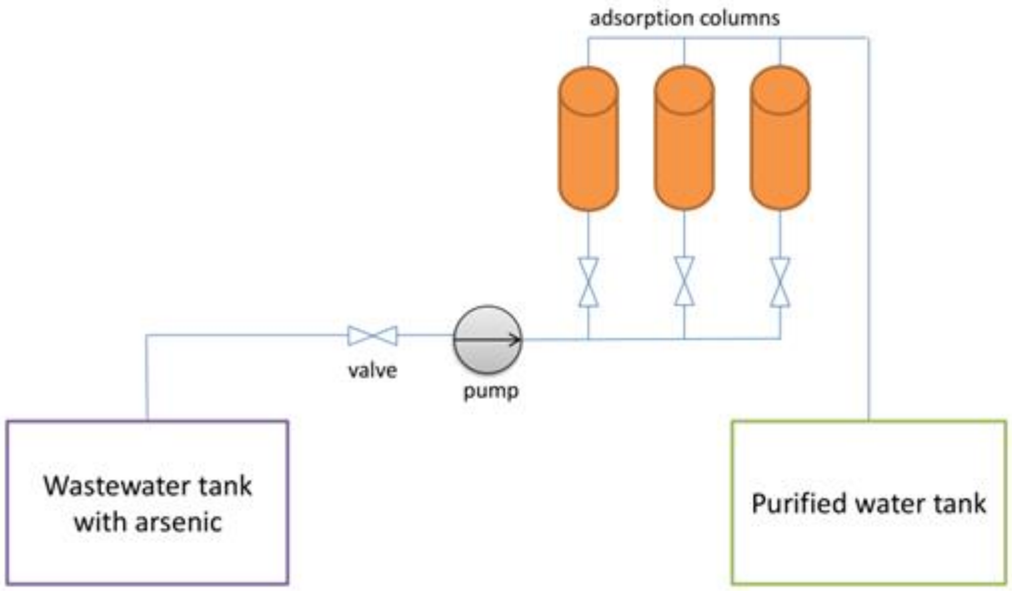

Fig.S3 Adsorption capacity at different amount of Fe-PE

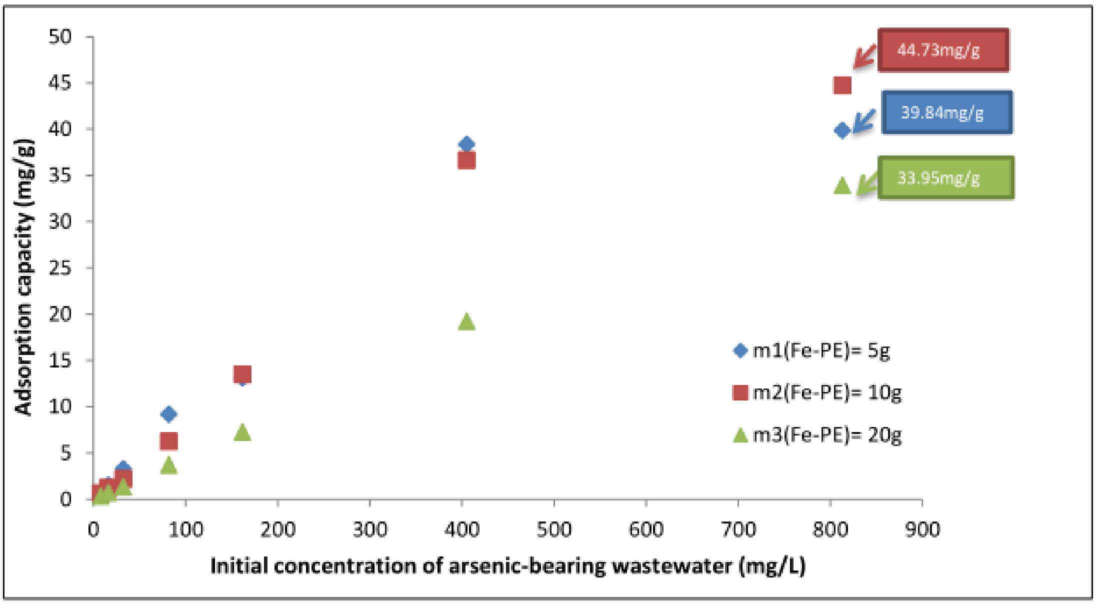

Fig.S4 Dissolution of Fe ions from Fe-PE in de-ionized water with different pH and the effect on arsenic removal efficiency of Fe-PE

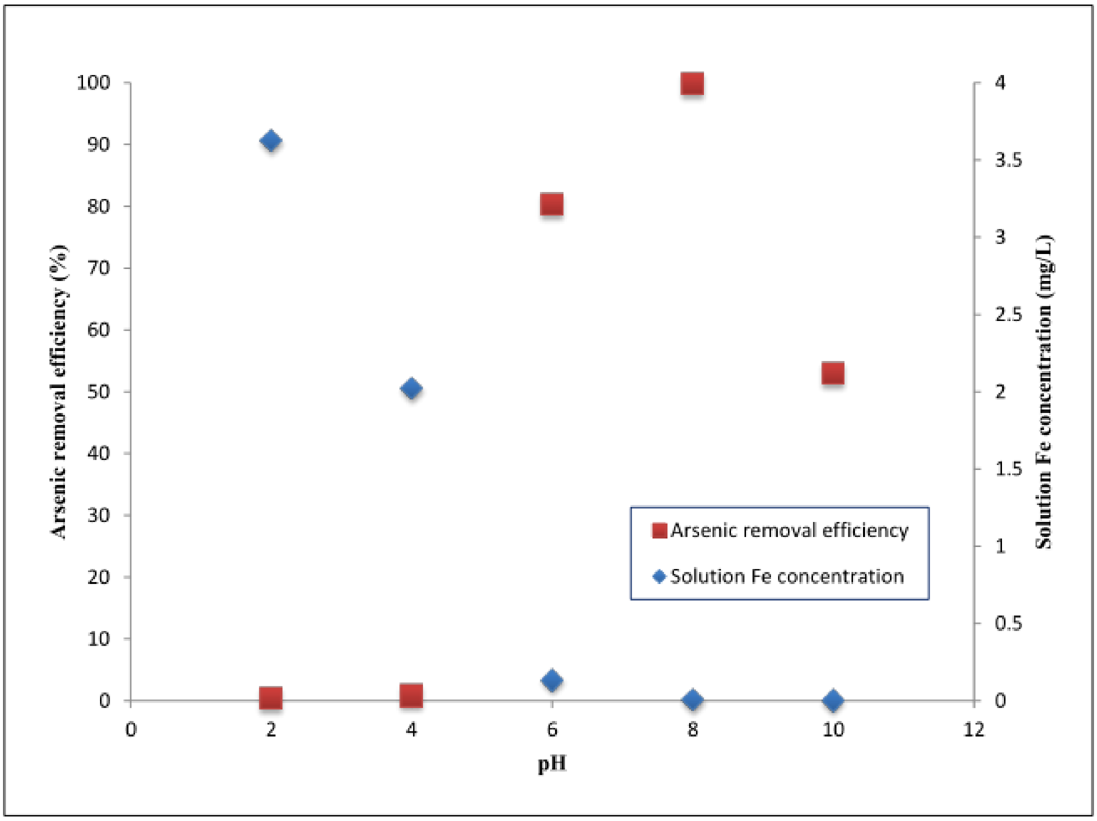

Fig.S5 Adsorption performance of Fe-PE before and after backflushing

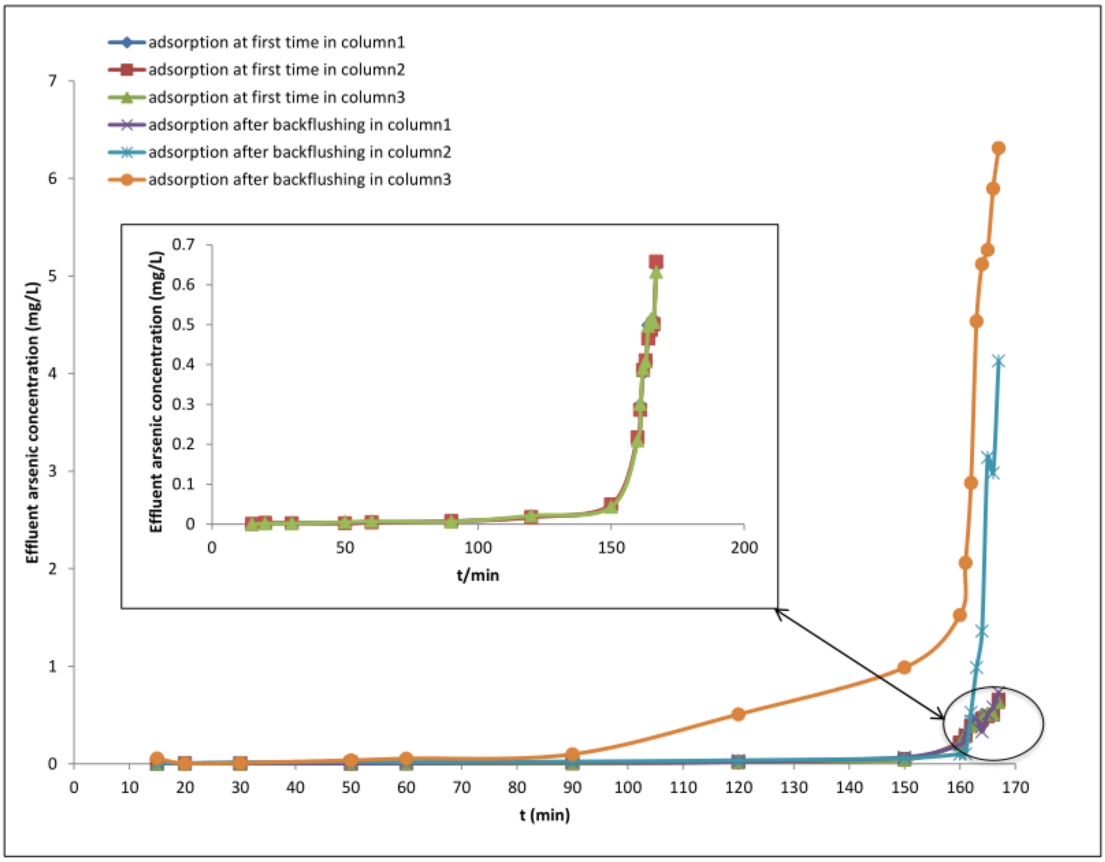

Method 1: Method of measuring the tensile resistance of AMFR

A Labthink XLW (L) PC auto-tensile tester was used for testing the stretching resistance of AMFR. Samples were dried at 30°C in the air oven and 60 pieces of AMFR were tested for average calculation.

CAUTION: (i) Researcher who makes experiments should wear lab coat, rubber shoes, respirator, gloves and protective goggles. (ii) The laboratory should run the mechanical ventilation to keep dry and ventilated. (iii) Operating the pilot equipment strictly according to the operating rules. It is strictly forbidden to change the operation order at will. The leak should be reported in time and the operation of the device should be suspended. (iv) Avoiding the skin to contact with the wastewater. if the skin inadvertently comes into contact with the wastewater, you should wash it with soap in time. If you inadvertently inhale the wastewater, vomiting or gastric lavage should be carried out, and 5% sodium dimercaptopropanesulfonate should be injected intramuscularly. (v) The symptoms of acute arsenic poisoning are nausea, vomiting, metallic taste in the mouth, severe abdominal pain, rice soup-like feces, etc.. The more severe ones are decreased urine volume, dizziness, gastrocnemius spasm, cyanosis and even shock, and in severe cases there are symptoms of central nerve paralysis. painful spasm of limbs, loss of consciousness and so on. Note: skin cancer is associated with arsenic intake and exposure, and lung cancer is associated with inhaled arsenic dust.

References

1. Regulations of the People's Republic of China on urban drainage and sewage treatment. 2014.01.01.
2. Ministry of Ecology and Environment, PRC. Announcement on the publication of the list of toxic and harmful Water pollutants (the first batch). 2019. 08.01.
3. Wang G J. Handbook of chemical raw materials: inorganic chemical raw materials. Chemical Industry Press, 2008: 630-631.
4. Wang X P, Wang Y T, Li Y S. Great dictionary of hygiene. Qingdao Publishing House. 2000.07.
